# Emotional Intelligence in the Professional Development of Nurses: From Training to the Improvement of Healthcare Quality

**DOI:** 10.3390/nursrep15080275

**Published:** 2025-07-30

**Authors:** Efthymia Chatzidimitriou, Sotiria Triantari, Ioannis Zervas

**Affiliations:** 1 Department of Early Childhood Education, University of Western Macedonia, 53100 Florina, Greece; 2 Department of International and European Studies, University of Macedonia, 54636 Thessaloniki, Greece; striantari@uom.edu.gr; 3 Department of Applied Informatics, University of Macedonia, 54636 Thessaloniki, Greece

**Keywords:** emotional intelligence, health workforce, ethics, nursing, professional competence, disaster planning, decision-making

## Abstract

**Background/Objectives**: Emotional intelligence has emerged as a key factor in shaping nursing performance and care quality, yet its specific mechanisms and impact within the Greek public healthcare context remain underexplored. This study aimed to investigate the role of emotional intelligence in ethical behavior, crisis management, and the perceived quality of care among nurses working in Greek public hospitals. **Methods**: A cross-sectional survey was conducted among practicing nurses using validated instruments to assess emotional intelligence, ethical compliance, crisis management skills, and care quality. Data were analyzed using covariance-based structural equation modeling (CB SEM) to examine both direct and indirect relationships among variables. **Results**: The results indicated that emotional intelligence training had a strong and significant effect on nurses’ ethical behavior and their ability to manage critical situations. However, the direct effect of emotional intelligence on the perceived quality of care was not significant; instead, its influence was mediated through improvements in ethics and crisis management. **Conclusions**: These findings suggest that the benefits of emotional intelligence in nursing are most evident when integrated with supportive organizational practices and ongoing professional development. Overall, this study highlights the need for comprehensive emotional intelligence training and a supportive workplace culture to enhance ethical standards, resilience, and patient care quality in Greek healthcare settings.

## 1. Introduction

The COVID-19 pandemic posed unprecedented challenges to healthcare and brought new psychological and professional pressures for nurses, placing them at the frontline of care [1]. Over the past decades, nurses have shown an ability to adapt, solve problems, and build psychological resilience, especially through training in emotional intelligence (EI) [2]. Goleman defined EI as “the capacity for recognizing our own feelings and those of others, for managing emotions well, and for motivating ourselves” [2]. Participation in EI training is expected to help nurses develop empathy, active listening, and communication—skills central to high-quality healthcare [3].

Importantly, recent research has confirmed that emotional intelligence is crucial not only for managing stress but also for improving job performance and communication skills in nursing, especially during health crises [4,5].

Given this background, the present quantitative, cross-sectional study in the third Health Region of Greece investigates the links between emotional intelligence, ethical behavior, crisis management, and perceived care quality.

The purpose of this study is to examine, using a statistical analysis, four hypotheses: (a) the impact of education on EI, (b) the relationship between EI and ethical compliance, (c) the role of EI in crisis management, and (d) EI’s contribution to care quality and patient satisfaction. This research provides evidence-based data on the value of EI training and offers practical guidance for educators and clinical leaders.

This article produces new empirical data from Greek nursing care, which may support both nursing practice and policy for staff empowerment. Furthermore, it also highlights the need for further studies with larger samples and broader approaches.

There is a growing call for more research to identify how EI can be systematically developed and applied within different healthcare systems [4].

To provide a comprehensive theoretical background and highlight the relevance of emotional intelligence to modern nursing challenges, the following review of the literature focuses on four key domains: EI development and training, ethics and Codes of Conduct, crisis management, and healthcare quality and patient satisfaction.

This integrated review aims to situate the present research within the context of recent international and Greek studies and to clarify the conceptual framework underlying this study’s hypotheses.

First, the development of EI in nursing is approached through a variety of methods, including introspection, didactic lessons, skills-based training, and blended/hybrid educational programs [6,7,8,9,10,11]. Studies show that such programs—whether brief workshops or extensive courses—can foster self-awareness, self-regulation, empathy, social skills, and motivation. However, the duration and intensity of training vary greatly, and while many interventions report positive effects, questions remain about the sustainability and generalizability of these outcomes [8,12,13,14,15].

A central debate in the literature is whether EI is an innate trait or a skill that can be developed through education. Most evidence suggests that targeted programs—combining theoretical instruction and practical exercises—can enhance key EI components, although the magnitude and duration of these gains are sometimes modest and context-dependent [14,15,16].

Capacity-building remains a challenge in healthcare, as over 70% of organizations report “capacity gaps” in workforce readiness [17]. Therefore, to address emotional and psychological stress, it is vital for nurses to receive training that helps them cope with burnout and enhances overall job satisfaction [18,19].

Turning to the ethical dimension, the intensification of ethical dilemmas in nursing is closely tied to advances in technology, increased patient expectations, and complex care scenarios [20]. Nurses are often at the forefront of ethical decision-making, which requires balancing clinical guidelines, patient autonomy, and organizational values [21,22].

Emotional intelligence is increasingly recognized as a key factor that underpins moral sensitivity and ethical behavior [23,24,25,26]. EI fosters empathy, critical thinking, and self-awareness—all of which are essential for responding effectively to moral challenges and upholding professional conduct. Research demonstrates that higher EI is associated with improved ethical decision-making, though this link is sometimes moderated by the workplace culture and support systems [27,28].

Furthermore, moral sensitivity—supported by emotion, empathy, and critical reflection—enables nurses to act in the best interests of patients even in ambiguous or difficult situations. Nonetheless, the relationship between EI and ethics is complex, and further study is needed to understand the interplay of personal, organizational, and contextual factors [27,29,30].

With regard to crisis management, the ability to manage crises—whether arising from health emergencies, natural disasters, or systemic failures—depends on effective planning, resilience, and emotional regulation [31,32,33,34]. EI plays a pivotal role by equipping nurses with the skills to remain calm, make sound decisions, and foster teamwork under pressure [35,36].

Crisis management training that incorporates EI principles has been shown to reduce stress, enhance communication, and improve outcomes for both staff and patients. However, evidence suggests that organizational factors (such as leadership, communication structures, and resource availability) are equally critical in determining the crisis response effectiveness [37,38].

Despite the recognized value of EI, more empirical studies are needed to clarify its unique contribution to crisis management, particularly in diverse healthcare systems.

Regarding quality and satisfaction, numerous studies confirm that nurses’ emotional competencies are closely linked to healthcare quality, patient satisfaction, and workforce stability [29,39,40,41,42,43]. High EI supports psychological empowerment, resilience, and better interpersonal communication, which in turn can improve clinical outcomes and enhance the patient experience [41,42].

Nevertheless, burnout remains a persistent threat, undermining care quality and professional well-being [40,44]. Programs designed to build EI can mitigate these risks, but the literature also points to measurement challenges and inconsistent findings regarding the direct effects of EI on patient satisfaction [45,46].

In response to these gaps, this study investigates how EI influences ethical decision-making, crisis management, and the quality of care in Greek public hospitals.

Nevertheless, recent systematic reviews and randomized controlled studies provide increasingly robust evidence for the value of emotional intelligence interventions in nursing. New data demonstrate that EI training programs can improve resilience, communication skills, and psychological well-being for both nursing students and professionals [47,48]. These findings reinforce the need for systematic EI development in nursing education and practice.

The Greek public nursing service is closely interdependent with society, the economy, science, and education, and faces increasing pressure to improve healthcare provision, organization, and processes. The economic recession has brought significant socio-economic challenges, while reduced purchasing power has increased demand for public care, adding to the workload of public health staff [48,49]. As a result, nurses are at greater risk of work burnout [49,50]. Health regions are called to use their available human resources as efficiently as possible. Nurses are vital for smooth operation of nursing services, and their ongoing education is essential.

Based on the identified research gaps, the following research questions were formulated:

RQ 1: How effectively can emotional intelligence skills be improved through training programs for nursing staff?

RQ 2: To what extent does the emotional intelligence of nursing staff affect their compliance with ethical principles and patients’ rights?

RQ 3: How does emotional intelligence affect nursing staff’s ability to manage critical situations (e.g., emergencies)?

RQ 4: How does the emotional intelligence of nursing staff affect the quality of care provided and patient satisfaction?

Addressing these questions, this study not only fills important gaps in the literature but also informs policy and training for Greek public health services.

### Conceptual Model

**Figure 1:**
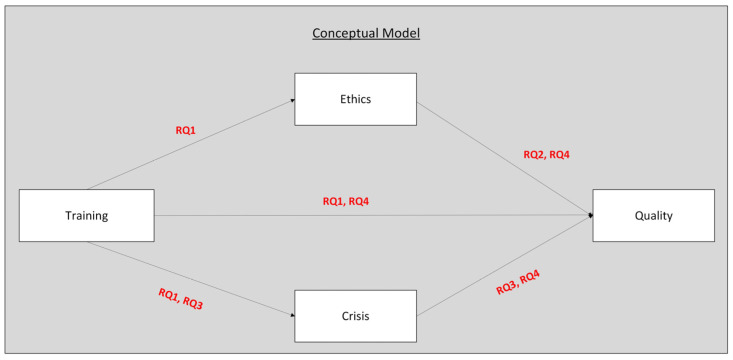
Conceptual model of study variables.

The conceptual model for this research is illustrated in Figure 1. This model tries to give a clear overview of the main variables and the expected relations between them, based on the research questions.

From the training construct, there are direct paths leading to ethics and to crisis management, as well as a direct connection with the quality of care and patient satisfaction. The path from training to ethics reflects the first research question (RQ1) about how much emotional intelligence can be improved through training and if this improvement can help nurses act more ethically and respect patients’ rights (RQ2). At the same time, training is hypothesized to improve crisis management abilities (RQ3), since emotional intelligence skills are important for managing stress and emergencies.

Ethics is connected with quality, because ethical nursing practice is closely related to better patient care and satisfaction (RQ2, RQ4). Also, crisis management is linked to quality (RQ3, RQ4), showing that nurses who handle critical situations effectively can also offer a higher quality of services. At the same time, training is expected to have a direct influence on quality (RQ1, RQ4) but also indirect effects through both ethics and crisis management. This shows that the effect of training is not always direct but may work through these other dimensions.

Each arrow in the model has a label (RQ1, RQ2, etc.) to show which research question it addresses. This helps to connect the visual model with the actual study aims and makes the logic of the research easier to understand. The structure of the model follows recommendations from nursing research, where conceptual frameworks are used to clarify complex relationships between variables and guide both the hypotheses and the analysis [51].

In summary, the model aims to capture the complexity of nursing practice in Greek public hospitals, where emotional intelligence, ethics, crisis responses, and quality are all closely connected. By mapping the research questions onto this structure, this study hopes to provide not just statistical results but a more practical understanding of how training in emotional intelligence can contribute to better outcomes for both nurses and patients.

## 2. Materials and Methods

### 2.1. Study Design and Setting

This study adopted a quantitative, cross-sectional design, and the survey was conducted fully online using Microsoft Forms (Office 365), which made it much easier to reach the nurses. The research period was from 15 January to 31 March 2025, focusing only on nursing staff in hospitals and healthcare units of the 3rd Regional Health Authority in Northern Greece, mostly in Thessaloniki.

### 2.2. Data Collection Procedure

A total of 1875 invitations were distributed via email through the human resources departments, and 407 nurses completed the survey, resulting in an overall response rate of 21.7%.

### 2.3. Sampling

Convenience sampling was chosen as the only realistic option given the hospital conditions and the high workload of nurses, making random sampling practically impossible. This approach is commonly used in healthcare research, although it introduces the risk of bias and limits the generalizability of results [52,53]. Despite this limitation, our aim was to collect representative data from those willing and available to participate.

### 2.4. Measurements

Following the sampling procedure, attention turns to the tools used for data collection. The questionnaire was structured in to be easy and not tiring for the participants, with the average time to complete it estimated between 28 and 31 min, depending on the personal pace of each respondent. It was divided into two main sections. The first part included five questions about demographic characteristics, such as gender, age, education level, years of experience, and current job position, aiming to describe the sample’s basic features.

The second section was the core part of the survey and was organized around four main thematic axes, with each axis consisting of eight questions. The first axis explored issues related to education and development of emotional intelligence, the second focused on ethics, Code of Conduct, and emotional intelligence in the nursing service, the third axis examined the role of emotional intelligence in crisis management, and the fourth covered emotional intelligence, quality of care, and patient satisfaction. All questions in this part used a five-point Likert scale, ranging from 1 (“Strongly Disagree”) to 5 (“Strongly Agree”), allowing participants to express the degree to which they agreed with each statement.

A detailed overview of all four thematic axes and their corresponding items is provided in the Results section (see Table 1).

### 2.5. Questionnaire Development and Content Validation

Τhe development of the questionnaire items was based on a comprehensive review of the international literature on emotional intelligence, nursing ethics, crisis management, and quality of care [2,4,47,54,55].

For content validation, the preliminary version was reviewed by an expert panel (two university professors and two nurse managers) who assessed the clarity and relevance of each item. Based on their feedback, minor improvements were made in item wording and structure. The questionnaire was then pilot tested with 15 nurses to further check clarity and usability, leading to additional minor revisions. However, no structured quantitative method for content validity assessment was used, which is a methodological limitation of this study [56,57].

### 2.6. Statistical Analysis

With the data collection instruments in place, appropriate statistical analyses were conducted to test the research hypotheses and evaluate the measurement model. For the main statistical analysis, we used covariance-based structural equation modeling (CB SEM). This method was chosen because it allows the researcher to test complex models with multiple variables and to estimate both direct and indirect effects between them at the same time. CB SEM is especially useful when the study includes latent variables, like emotional intelligence or quality of care, which are measured through several survey items. Another important advantage is that it provides good tools for testing the overall fit of the model and for checking the validity and reliability of the constructs [58,59].

To examine both direct and indirect effects among the latent variables, mediation analysis was conducted within the CB-SEM framework. The statistical significance of all direct and indirect (mediation) effects was assessed using bootstrapping procedures (5000 resamples), following recent recommendations in SEM literature [60,61]. For each specific mediation path, estimates of effect size, standard error (SE), *t*-value, and *p*-value were obtained. Bootstrapping was selected as the preferred method because it provides more robust confidence intervals for indirect effects than traditional tests like the Sobel test.

The following specific mediation paths were tested:

1. Training → Ethics → Quality of Care
2. Training → Crisis Management → Quality of Care

To capture each latent variable in the SEM model, we used groups of eight questionnaire items that corresponded to the four main theoretical constructs. The first group of items referred to education and development of emotional intelligence, the second covered ethics and Code of Conduct in nursing, the third related to emotional intelligence in crisis management, and the last group focused on quality of care and patient satisfaction. All items were measured reflectively with five-point Likert scales, ensuring that each dimension of the construct was assessed from multiple angles. The assignment of item codes and a brief description of the content for each question is presented in Table 1.

Reliability and validity of the measurement model were evaluated using several established statistical indicators. Internal consistency was assessed with Cronbach’s alpha and Composite Reliability (CR) for each latent construct. Convergent validity was examined by calculating the Average Variance Extracted (AVE) for every construct, while discriminant validity was tested using both the Fornell–Larcker criterion and the Heterotrait–Monotrait (HTMT) ratio, as recommended in the SEM literature [58,62,63].

All analyses were performed using SmartPLS (version 4.1.1.4).

### 2.7. Model Fit and Bias Assessment

Model fit was assessed using several established indices, including the Comparative Fit Index (CFI), Tucker–Lewis Index (TLI), Root Mean Square Error of Approximation (RMSEA), Standardized Root Mean Square Residual (SRMR), and chi-square test, following recommended guidelines [64]. Additional indices such as NFI, GFI, AGFI, PGFI, AIC, and BIC were also examined.

Common method bias was tested with Harman’s single-factor test, by including all measurement items in an exploratory factor analysis without rotation [65].

Detailed fit indices, loadings, and bias results are reported in the Results and Appendix A (Table A1, Table A2 and Table A3).

### 2.8. Ethical Considerations

Ethical approval for this study was obtained from both the Ethics Committee of the 3rd Regional Health Authority (Protocol No 14329/2025) and the Ethics Committee of the University of Western Macedonia (Protocol No 244/2025), in full compliance with the Declaration of Helsinki and GDPR (EU 2016/679). Participation was entirely voluntary; only registered nurses or nurse managers working in the 3rd Regional Health Authority during the research period (15 January to 31 March 2025) and aged 18 years or older were eligible. All participants were informed in detail about the purpose of this study, their rights, and data protection measures, and explicit informed consent was obtained electronically before they could access the questionnaire. No sensitive personal data were collected, all responses were fully anonymized, and the dataset generated and analyzed during the study is openly available at https://doi.org/10.6084/m9.figshare.29425484, (accessed on 20 June 2025).

## 3. Results

### 3.1. Descriptive Statistics

**Figure A1:**
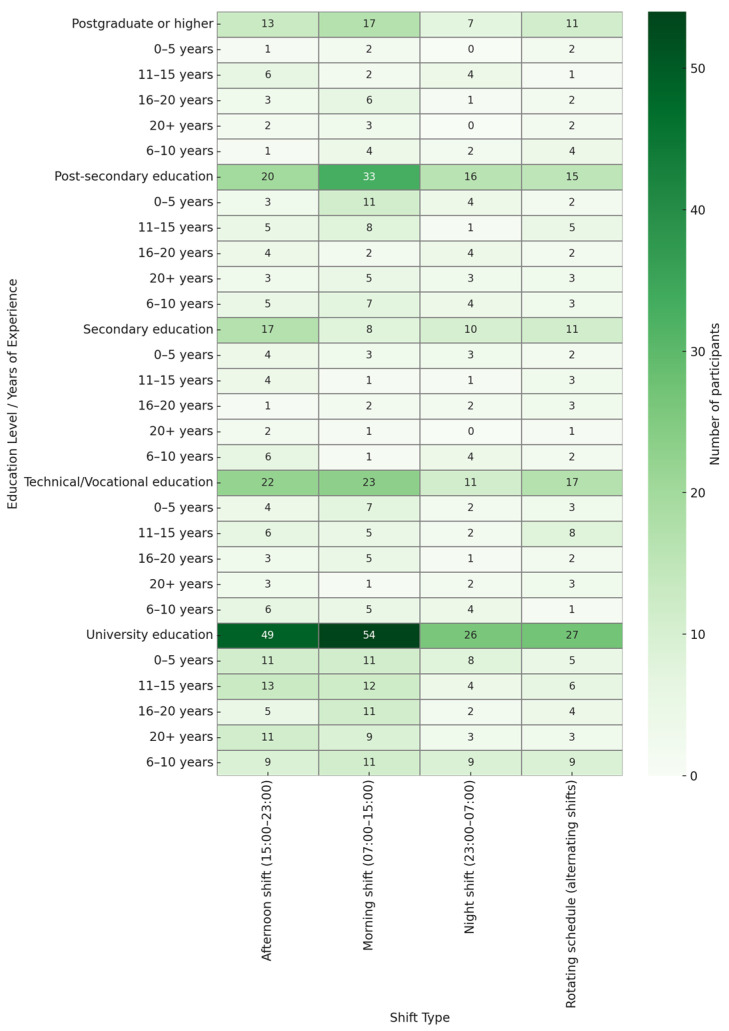
A heatmap showing the distribution of shift schedules by education level and work experience. University-educated nurses are most often scheduled for morning and afternoon shifts, whereas those with post-secondary or technical/vocational training are more evenly distributed across all shifts. Nurses with up to 10 years of service are mainly found in morning and afternoon shifts, while those with longer tenures are more likely to work night or rotating shifts. This pattern is consistent with previous empirical evidence [87,88,89].

**Figure A2:**
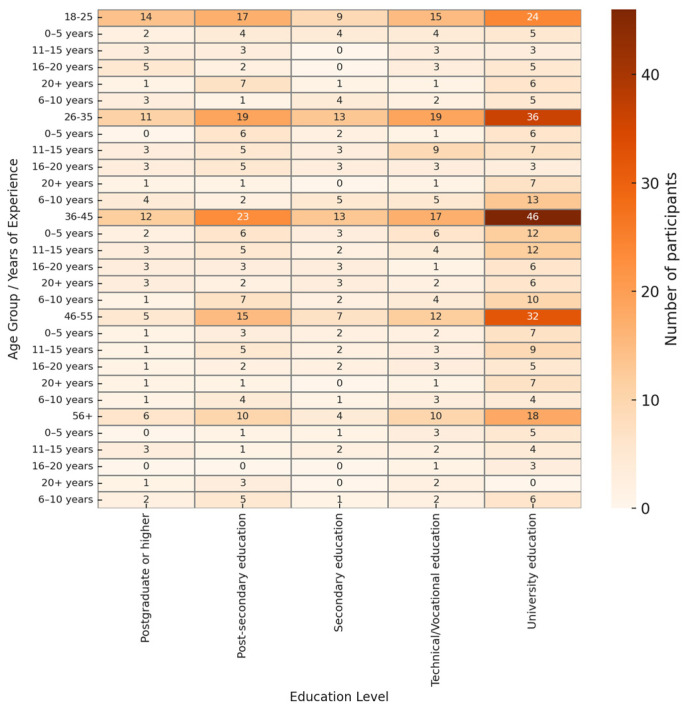
A heatmap of ages and education among the sample. The most populated groups are nurses aged 26–45 years with university or post-secondary education, often combining 6–15 years of work experience. Older nurses and those with more than 20 years of experience, especially with postgraduate qualifications, are less common, a finding consistent with other European studies [90,91,92].

Sociodemographic characteristics of the sample are reported in Appendix A (Figure A1 and Figure A2), where heatmaps of the age, gender, education, work experience, and job position were generated using Python (v3.10.12) and the scientific libraries pandas, matplotlib, and seaborn, following current best practices [66,67].

The main sections and items of the questionnaire are presented in Table 1.

### 3.2. Measurement Model and Model Fit

The internal consistency reliability for all constructs was excellent, with Cronbach’s alpha and Composite Reliability (CR) values well above the accepted threshold of 0.70. The convergent validity was confirmed as all Average Variance Extracted (AVE) values exceeded 0.60, and discriminant validity was supported by both HTMT ratios (<0.84) and Fornell–Larcker criteria. Full details and indicator values are provided in Appendix A, Table A4, Table A5 and Table A6.

The estimated model showed an excellent fit to the data, with a CFI = 0.997, TLI = 0.996, RMSEA = 0.014, and SRMR = 0.033, all well within recommended thresholds [64]. The full set of model fit indices is available in Appendix A, Table A1.

The chi-square statistic (χ^2^ = 497.672, df = 459, *p* = 0.103) and the chi-square/df ratio (1.084) also confirmed the very good model fit. The full set of fit indices is reported in Appendix A, Table A1. All item loadings on their respective constructs were high and statistically significant (all > 0.91, *p* < 0.001), supporting convergent validity (Appendix A, Table A2). A common method bias was not detected, as the first factor explained only 42% of the variance, below the 50% threshold (Appendix A, Table A3) [65].

### 3.3. Structural Model Results

**Figure 2:**
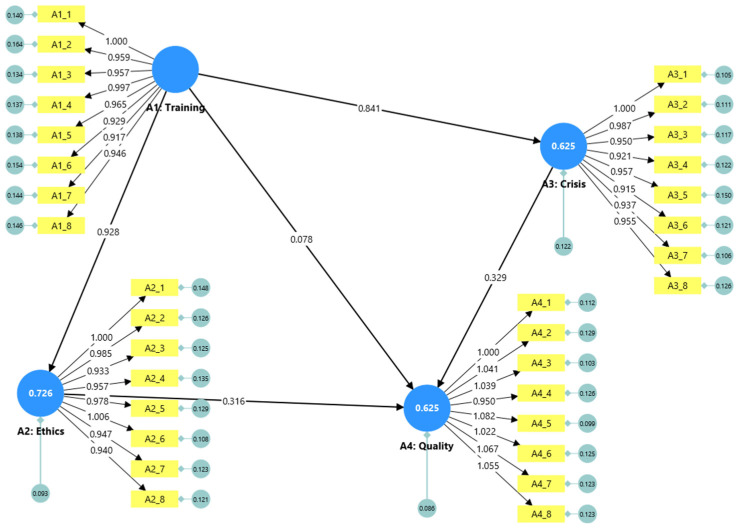
SEM path diagram.

Figure 2 illustrates the main structural paths of the CB-SEM model, with standardized coefficients and significance levels. The path from training to ethics is very strong and statistically significant, as is the path from training to crisis management. In contrast, the direct effect of training on the quality of care is small and not significant, while the paths from ethics and from crisis management to quality of care are both moderate and statistically significant, as indicated in Figure 2.

### 3.4. Mediation Analysis

The results of the mediation analysis are presented in Table 2, which combines all direct, specific indirect, total indirect, and total effects with their 95% bootstrap confidence intervals. The effect of training on the quality of care was mainly indirect, mediated through both ethics (specific indirect effect = 0.293, *p* < 0.001) and crisis management (specific indirect effect = 0.277, *p* < 0.001). The total indirect effect of training on the quality of care was 0.570 (*p* < 0.001), while the direct effect was not significant (0.078, *p* = 0.371). The total effect, including all pathways, was 0.648 (*p* < 0.001), showing that training remains a main predictor of the perceived care quality mostly through these mediators.

All effects and their statistical significance were calculated with bootstrapping (5000 samples), as described in the Methods. These findings agree with other nursing research that highlights how professional development builds both ethical and crisis management skills, which are essential for a higher care quality [27].

### 3.5. Research Questions

#### 3.5.1. RQ1: Improvement of Emotional Intelligence Skills

RQ1 was assessed using CB-SEM, evaluating the effect of emotional intelligence (EI) training on the improvement of EI skills among nursing staff.

The analysis revealed a very strong and statistically significant effect (β = 0.928, *p* < 0.001), indicating that systematic training substantially enhances nurses’ emotional intelligence skills. This finding is consistent with previous studies showing the effectiveness of EI-focused educational programs in nursing [27].

#### 3.5.2. RQ2: EI and Ethical Standards

RQ2 was assessed using CB-SEM to examine the association between emotional intelligence and compliance with ethical standards and patients’ rights. The structural path from EI training to ethics was strong and significant (β = 0.928, *p* < 0.001). Nurses with higher emotional intelligence, enhanced through training, demonstrated a greater adherence to ethical principles and the protection of patient rights. This result aligns with research linking emotional intelligence to ethical conduct in nursing [18,49].

#### 3.5.3. RQ3: EI and Crisis Management

RQ3 was assessed using CB-SEM to test the effect of emotional intelligence on crisis management skills among nursing staff. Results indicated a strong and significant effect of EI training on the ability to manage critical situations (β = 0.841, *p* < 0.001). Nurses who participated in EI training were better equipped to handle emergencies and stressful conditions, as supported by recent meta-analyses on EI interventions [21].

#### 3.5.4. RQ4: EI, Quality of Care, and Patient Satisfaction

RQ4 was assessed using CB-SEM with a mediation analysis to examine both the direct and indirect effects of emotional intelligence on the quality of care and patient satisfaction. The direct effect of EI training on quality of care was small and not statistically significant (β = 0.078, *p* = 0.303). However, there were strong indirect effects through the mediators of ethics (β = 0.316, *p* < 0.001) and crisis management (β = 0.329, *p* < 0.001). This suggests that improvements in nurses’ emotional intelligence primarily influence care quality and patient satisfaction indirectly, through strengthened ethical behavior and crisis management skills. This mediation pattern is supported by previous findings in the field [20,28].

## 4. Discussion

The present findings clarify the links between emotional intelligence training, ethics, crisis management, and nursing care quality in Greek hospitals. The outcomes of the structural equation model are discussed per research question and compared with the recent literature.

Recent studies further highlight the importance of emotional intelligence in nursing. Large-scale meta-analyses and intervention studies consistently show that EI training significantly improves nurses’ ethical sensitivity, decision-making, and resilience, especially in demanding clinical environments [4,5,12]. Importantly, new evidence suggests that EI benefits patient care mainly through indirect effects—such as enhanced ethical practice and crisis management—rather than direct improvement of patient satisfaction [10]. The experience of the COVID-19 pandemic has underscored the urgent need for systematic EI development, both in education and ongoing professional development, to support both staff well-being and care quality [68]. Our results align with and expand on these findings, confirming the centrality of emotional intelligence in nursing practice.

This study addressed four main research questions. For RQ1, the results confirmed that systematic training programs can significantly enhance nurses’ emotional intelligence skills (β = 0.928, *p* < 0.001), supporting prior evidence for the effectiveness of targeted EI interventions [27,37].

Regarding RQ2, the findings demonstrated that improved EI, as a result of training, is strongly associated with an adherence to ethical principles and patient rights, in line with international research linking EI to ethical behavior in nursing [27,69,70].

For RQ3, the analysis showed that emotional intelligence has a significant positive impact on nurses’ ability to manage critical situations, confirming the value of EI for resilience and crisis management (β = 0.841, *p* < 0.001) [71].

In relation to RQ4, the direct effects of EI training on quality of care were not statistically significant (β = 0.078, *p* = 0.303). However, strong indirect effects were found through the mediators of ethics (β = 0.316, *p* < 0.001) and crisis management (β = 0.329, *p* < 0.001), indicating that the pathway from EI to care quality is primarily indirect—which is consistent with previous research [72,73].

Overall, the results confirm the importance of integrating ethics and crisis management into nurse professional development programs, as these areas maximize the positive impact of EI training on care quality and patient satisfaction.

These findings are consistent with international studies, which associate higher emotional intelligence in nursing with improved patient satisfaction and care outcomes, often through the mediation by ethics and crisis management. For instance, researchers found positive links between nurses’ EI, therapeutic relationships, and patient satisfaction, especially when emotional skills are embedded in everyday practice [70,71].

### 4.1. Practical Implications

The findings of this study offer several actionable implications for nursing practice, education, and healthcare management. First, integrating emotional intelligence (EI) training into both undergraduate and continuing professional development can yield significant benefits. Programs emphasizing communication, empathy, resilience, and particularly ethical decision-making and crisis management—using interactive methods such as simulation and reflective exercises—are recommended, as these are the main pathways through which EI impacts care quality [4,74].

Second, healthcare organizations should value EI not only for individual performance but as a foundation for compassionate, values-based care. Evaluating EI as part of staff assessments and linking it to outcomes like patient satisfaction and safety can support a more patient-centered culture [74,75].

Third, higher EI among nurses is associated with improved patient safety, the better detection of needs, more effective teamwork, and reduced adverse events [76]. Investing in emotional skills thus supports both care quality and workforce sustainability, helping prevent burnout and staff turnover [72].

Fourth, organizational EI strategies—including teamwork, open communication, and structured debriefings—can help healthcare environments adapt to crises and maintain high standards of care. Nurse leaders should model and reinforce these behaviors [74,77].

Fifth, policy should encourage innovation in EI assessment and intervention, including piloting new training models, using digital self-assessment tools, and supporting research on long-term outcomes [74]. Blended training formats, combining in-person and online learning, and ongoing support mechanisms, like mentoring and feedback sessions, can help sustain gains from EI interventions [73,76,78].

Finally, to maintain the positive impact of EI training, regular reinforcement through follow-up sessions and continuous professional development is necessary. Future research should examine the sustainability of training effects and identify effective strategies for skill retention [79,80].

In summary, an integrated approach that combines EI training, organizational support, and regular evaluation can improve both patient outcomes and staff well-being, providing a basis for more resilient and effective healthcare services.

### 4.2. Theoretical Implications

This study advances the theoretical understanding of emotional intelligence (EI) in nursing by empirically confirming its multidimensional impact on clinical practice. Our findings show that EI functions not only at the personal level—supporting empathy, resilience, and communication—but also shapes ethical decision-making and crisis management skills among nurses [70,74]. This suggests that EI is a dynamic construct supporting both intrapersonal and interpersonal functioning in healthcare.

The recent evidence from randomized trials and global reviews demonstrates that targeted EI interventions improve nurses’ resilience, communication, stress management, and organizational outcomes, while also reducing burnout and enhancing teamwork [47,48]. These results underline the value of EI development programs, which is consistent with our study’s conclusions.

Importantly, our model indicates that EI’s impact on care quality and patient satisfaction is mainly indirect, operating through ethical behavior and crisis management skills. This highlights the importance of mediation mechanisms, rather than direct effects, in the EI–outcome relationship [75]. The results support a total mediation model, showing that training affects care quality almost exclusively through improvements in ethics and crisis management. Thus, these competencies are key channels through which training influences patient outcomes.

Finally, our findings point to the role of organizational and contextual factors in shaping how EI is expressed and utilized. The Greek public hospital setting, with its particular challenges, illustrates the need to adapt theoretical models to local contexts. Future research should consider the organizational culture, resources, and policy to better understand the antecedents and effects of EI in nursing [13,49,74].

### 4.3. Limitations and Future Research

Despite the methodological rigor of this study, several limitations should be considered when interpreting the findings.

First, the sample was drawn using a convenience approach from a single regional health authority. While this method was practical given resource constraints, it can introduce selection bias, as nurses who chose to participate may have different attitudes or skills than those who did not [53]. This means the findings may not fully represent all nurses in Greece or similar international settings.

Second, because the study design was cross-sectional, we cannot determine the direction of relationships between emotional intelligence, ethics, crisis management, and care quality. All data were collected at a single point in time, making it difficult to know which factors influence each other over time [81]. Moreover, the assumed causal structure in the SEM model (Training → Mediators → Quality) may not fully capture the real-world dynamics, which are likely to be more reciprocal or bidirectional. Future research could use longitudinal or cross-lagged panel designs to better explore the directionality and potential reciprocal influences among these constructs over time.

Third, the exclusive use of self-report questionnaires is a common limitation in behavioral research. Although widely accepted, this method is prone to biases such as social desirability and recall errors, even when statistical checks like Harman’s test do not suggest major problems [65]. Using data from multiple sources or applying more advanced controls in future research could help reduce such bias.

Fourth, the external validity of this study is limited because it was conducted in a specific healthcare context. Different regions, organizations, or patient populations may face unique challenges, and our findings may not apply everywhere [51,82]. Comparative or multi-site studies could help to better generalize results.

Fifth, relying solely on quantitative self-report data may not capture the full complexity of how emotional intelligence appears in everyday nursing practice [83,84]. Including interviews, observations, or mixed methods in future research could offer a richer understanding of these dynamics.

Additionally, the questionnaire developed for this study was based on a thorough literature review and pilot testing, but it may not include every aspect of emotional intelligence, and it has not yet been validated in other settings. This could limit the precision and applicability of the results.

Although the sample size (*n* = 407) was adequate according to SEM guidelines, the complexity of the model increases the risk of overfitting. Using larger samples or simplifying the model could strengthen the robustness of future findings [85,86].

It is also worth noting that the strong association observed between the ethics and crisis management constructs may reflect some conceptual overlap, a common issue in studies with related psychological variables. Advanced statistical approaches, such as higher-order or bifactor modeling, may help clarify these relationships in future work.

A final limitation is that the presence of some factor loadings greater than 1.0 (Heywood cases) could reflect item redundancy or multicollinearity within the constructs. Although no indicator had to be removed due to low loading in the current study, future research may benefit from item reduction, the re-examination of item distinctiveness, or alternative modeling strategies to minimize such effects.

Overall, addressing these limitations—by adopting probabilistic sampling and longitudinal and mixed-method designs, collecting data from multiple sites, and triangulating data sources—will help strengthen the quality and generalizability of future research. These improvements can provide deeper insights into how emotional intelligence shapes nursing practice and patient care.

## 5. Conclusions

This study demonstrates that emotional intelligence is a key aspect of nursing practice, shaping ethical behavior, crisis management, and, indirectly, patient care quality in Greek public hospitals. The findings provide evidence that targeted emotional intelligence training can enhance both individual skills and organizational outcomes. Results support the integration of emotional skills into ongoing nurse education and highlight the multifaceted effects of emotional intelligence on personal and professional levels.

Moreover, the research shows that the benefits of emotional intelligence are maximized in supportive workplace cultures with strong ethical frameworks. Although the direct link between emotional intelligence and care quality was limited, the mediation through ethics and crisis management highlights the need for comprehensive strategies to improve patient outcomes.

These results suggest clear priorities for policy and practice: invest in evidence-based emotional intelligence training, embed it in professional development, and promote environments that cultivate ethical conduct and emotional resilience. Implementing these recommendations may strengthen staff well-being and enhance the quality of patient care across the Greek healthcare sector.

## Figures and Tables

**Table 1:** Items of questionnaire.

Item Code	Main Description of Each Question
Education and Development of Emotional Intelligence (EI)	
1A	EI training improves stress management
1B	Training helps with emotion regulation
1C	EI training supports work relationships
1D	Training should include EI education
1E	Program can enhance patient empathy
1F	EI skills need ongoing learning
1G	EI reduces professional burnout
1H	Willingness to join EI training
Ethics, Code of Conduct, and Emotional Intelligence	
2A	EI helps keep ethical principles
2B	EI assists ethical decision-making
2C	High EI prevents rights violations
2D	Empathy supports nursing ethics
2E	EI supports professional behavior
2F	EI helps ethical compliance
2G	EI nurses protect patient rights
2H	Low EI leads to ethical dilemmas
Emotional Intelligence in Crisis Management	
3A	EI keeps calm in emergencies
3B	Anxiety control aids decisions
3C	Self-awareness helps ask for help
3D	Recognizing feelings in patients during crisis
3E	EI improves teamwork in emergencies
3F	High EI reduces errors under pressure
3G	EI defuses stressful situations
3H	EI is vital for crisis management
Emotional Intelligence, Quality of Care, and Patient Satisfaction	
4A	Empathy impacts care quality
4B	Patients respond to understanding nurses
4C	Self-regulation for politeness/professionalism
4D	EI builds patient trust
4E	Satisfaction when nurses listen and understand
4F	Understanding emotions enables personalized care
4G	Nurses’ EI affects patient satisfaction
4H	EI supports patience and professionalism

**Table 2:** Total standardized effects.

Pathway	Effect Type	O	M	STDEV	*t*	*p*	2.5%	97.5%
A1: Training → A2: Ethics	Direct	0.928	0.928	0.056	16.675	0.000	0.830	1.045
A1: Training → A3: Crisis	Direct	0.841	0.841	0.049	17.201	0.000	0.747	0.935
A2: Ethics → A4: Quality	Direct	0.316	0.313	0.077	4.119	0.000	0.149	0.472
A3: Crisis → A4: Quality	Direct	0.329	0.332	0.057	5.768	0.000	0.215	0.446
A1: Training → A4: Quality	Direct	0.078	0.078	0.087	0.895	0.371	−0.097	0.253
A1: Training → A3: Crisis → A4: Quality	Specific Indirect	0.277	0.279	0.050	5.553	0.000	0.180	0.385
A1: Training → A2: Ethics → A4: Quality	Specific Indirect	0.293	0.291	0.073	4.009	0.000	0.141	0.434
A1: Training → A4: Quality (Total Indirect)	Total Indirect	0.570	0.570	0.083	6.882	0.000	0.403	0.730
A1: Training → A4: Quality	Total Effect	0.648	0.647	0.043	15.153	0.000	0.566	0.733

## Data Availability

The original data presented in this study are openly available in https://doi.org/10.6084/m9.figshare.29425484, accessed on 20 June 2025.

## Public Involvement Statement

This study did not involve patients or members of the public in the design, conduct, reporting, or dissemination plans of our research.

## Guidelines and Standards Statement

This manuscript was drafted with reference to the STROBE guidelines for cross-sectional research.

## Appendix A

**Table A1:** Model fit indices.

	Estimated Model	Null Model
Chi-square	497.672	12,084.297
Number of model parameters	69.000	32.000
Number of observations	407.000	*n*/a
Degrees of freedom	459.000	496.000
*p* value	0.103	0.000
ChiSqr/df	1.084	24.364
RMSEA	0.014	0.240
RMSEA LOW 90% CI	0.000	0.236
RMSEA HIGH 90% CI	0.023	0.243
GFI	0.929	*n*/a
AGFI	0.919	*n*/a
PGFI	0.808	*n*/a
SRMR	0.033	*n*/a
NFI	0.959	*n*/a
TLI	0.996	*n*/a
CFI	0.997	*n*/a
AIC	635.672	*n*/a
BIC	912.280	*n*/a

**Table A2:** Outer loadings and significance levels for all measurement items.

	Parameter Estimates	Standard Errors	T Values	*p* Values
A1_1 <- A1: Training	1.000	*n*/a	*n*/a	*n*/a
A1_2 <- A1: Training	0.959	0.052	18.482	0.000
A1_3 <- A1: Training	0.957	0.049	19.460	0.000
A1_4 <- A1: Training	0.997	0.050	19.893	0.000
A1_5 <- A1: Training	0.965	0.050	19.429	0.000
A1_6 <- A1: Training	0.929	0.050	18.556	0.000
A1_7 <- A1: Training	0.917	0.049	18.718	0.000
A1_8 <- A1: Training	0.946	0.050	19.010	0.000
A2_1 <- A2: Ethics	1.000	*n*/a	*n*/a	*n*/a
A2_2 <- A2: Ethics	0.985	0.045	21.737	0.000
A2_3 <- A2: Ethics	0.933	0.044	21.176	0.000
A2_4 <- A2: Ethics	0.957	0.045	21.054	0.000
A2_5 <- A2: Ethics	0.978	0.045	21.545	0.000
A2_6 <- A2: Ethics	1.006	0.044	22.655	0.000
A2_7 <- A2: Ethics	0.947	0.044	21.455	0.000
A2_8 <- A2: Ethics	0.940	0.044	21.369	0.000
A3_1 <- A3: Crisis	1.000	*n*/a	*n*/a	*n*/a
A3_2 <- A3: Crisis	0.987	0.041	23.856	0.000
A3_3 <- A3: Crisis	0.950	0.041	22.934	0.000
A3_4 <- A3: Crisis	0.921	0.041	22.322	0.000
A3_5 <- A3: Crisis	0.957	0.044	21.597	0.000
A3_6 <- A3: Crisis	0.915	0.041	22.304	0.000
A3_7 <- A3: Crisis	0.937	0.040	23.441	0.000
A3_8 <- A3: Crisis	0.955	0.042	22.573	0.000
A4_1 <- A4: Quality	1.000	*n*/a	*n*/a	*n*/a
A4_2 <- A4: Quality	1.041	0.053	19.601	0.000
A4_3 <- A4: Quality	1.039	0.050	20.643	0.000
A4_4 <- A4: Quality	0.950	0.051	18.721	0.000
A4_5 <- A4: Quality	1.082	0.051	21.209	0.000
A4_6 <- A4: Quality	1.022	0.052	19.560	0.000
A4_7 <- A4: Quality	1.067	0.053	20.056	0.000
A4_8 <- A4: Quality	1.055	0.053	19.966	0.000

**Table A3:** Harman’s single-factor test.

Harman’s Single-Factor Test			
Factor	SS Loadings	% of Variance	Cumulative %
1	13.44	42.0	42.0

**Table A4:** Construct reliability and validity.

	Cronbach’s Alpha (Standardized)	Cronbach’s Alpha (Unstandardized)	Composite Reliability (Rho_c)	Average Variance Extracted (AVE)
A1: Training	0.937	0.936	0.936	0.645
A2: Ethics	0.953	0.952	0.953	0.715
A3: Crisis	0.952	0.951	0.952	0.711
A4: Quality	0.944	0.944	0.943	0.674

**Table A5:** Discriminant validity—Heterotrait–Monotrait ratio (HTMT).

	A1: Training	A2: Ethics	A3: Crisis	A4: Quality
A1: Training				
A2: Ethics	0.839			
A3: Crisis	0.774	0.763		
A4: Quality	0.709	0.748	0.742	

**Table A6:** Discriminant validity—Fornell–Larcker criterion.

	A1: Training	A2: Ethics	A3: Crisis	A4: Quality
A1: Training	0.803			
A2: Ethics	0.852	0.846		
A3: Crisis	0.791	0.674	0.843	
A4: Quality	0.726	0.724	0.721	0.821
